# Genetic polymorphisms in *TNF *genes and tuberculosis in North Indians

**DOI:** 10.1186/1471-2334-10-165

**Published:** 2010-06-10

**Authors:** Shilpy Sharma, Jaishriram Rathored, Balaram Ghosh, Surendra K Sharma

**Affiliations:** 1 Molecular Immunogenetics Laboratory, Institute of Genomics and Integrative Biology, Mall Road, Delhi-110007, India; 2 Department of Medicine, All India Institute of Medical Sciences, New Delhi-110029, India; 3 University of Michigan Medical School, Biomedical Science Research Building, 109 Zina Pitcher Place, Ann Arbor, MI 48109-2200, USA

## Abstract

**Background:**

Pulmonary tuberculosis, the most common clinical form of mycobacterial diseases, is a granulomatous disease of the lungs caused by *Mycobaterium tuberculosis*. A number of genes have been identified in studies of diverse origins to be important in tuberculosis. Of these, both tumor necrosis factor α (TNF-α) and lymphotoxin α (LT-α) play important immunoregulatory roles.

**Methods:**

To investigate the association of *TNF *polymorphisms with tuberculosis in the Asian Indians, we genotyped five potentially functional promoter polymorphisms in the *TNFA *gene and a *LTA_NcoI *polymorphism (+252 position) of the *LTA *gene in a clinically well-defined cohort of North-Indian patients with tuberculosis (N = 185) and their regional controls (N = 155). Serum TNF-α (sTNF-α) levels were measured and correlated with genotypes and haplotypes.

**Results:**

The comparison of the allele frequencies for the various loci investigated revealed no significant differences between the tuberculosis patients and controls. Also, when the patients were sub-grouped into minimal, moderately advanced and far advanced disease on the basis of chest radiographs, TST and the presence/absence of cavitary lesions, none of the polymorphisms showed a significant association with any of the patient sub-groups. Although a significant difference was observed in the serum TNF-α levels in the patients and the controls, none of the investigated polymorphisms were found to affect the sTNF-α levels. Interestingly, it was observed that patients with minimal severity were associated with lower log sTNF-α levels when compared to the patients with moderately advanced and far advanced severity. However, none of these differences were found to be statistically significant. Furthermore, when haplotypes were analyzed, no significant difference was observed.

**Conclusions:**

Thus, our findings exclude the *TNF *genes as major risk factor for tuberculosis in the North Indians.

## Background

Mycobacterial diseases are a major health concern, with an estimate of approximately one-third of the world's population being affected by them [1,2]. Pulmonary tuberculosis, the most common clinical form of the disease, is a granulomatous disease of the lungs caused by *Mycobaterium tuberculosis*. However, only 5-10% of the infected people ever develop the disease. The genetic contribution of the host plays a significant role in determining susceptibility to developing the active form of the disease, the severity of infection and the health outcome of the patient [3,4]. A number of genes have been identified in studies of diverse origins to be important in tuberculosis [5-7].

The genes for tumor necrosis factor-α (TNF-α; *TNFA*) and lymphotoxin-α (LT-α; *LTA*), located within the MHC III region of chromosome 6, shows close linkage to the HLA class I (*HLA-B*) and class II (*HLA-DR*) genes [8]. Both TNF-α, produced mainly by monocytes and activated macrophages; and LT-α, produced mainly by activated T-cells, play important immunoregulatory roles [9]. Of these, TNF-α contributes to the pathogenesis of tuberculosis due to its role in the formation and maintenance of granulomas [10]. Additionally, it also plays a major role in host defense to *M. tuberculosis *by its synergistic action with interferon-γ (IFN-γ) to activate macrophages and thereby impacts on disease perpetuation [11,12]. Elevated serum TNF-α (sTNF-α) levels have been reported in advanced tuberculosis patients when compared to those with mild tuberculosis and healthy individuals [13].

Studies on monozygotic twins and their first-degree relatives, using *ex vivo *endotoxin stimulated whole blood samples, have provided evidence that 60% of variation in the production capacity of TNF-α appears to be genetically determined [14]. Several polymorphisms within the promoter region of *TNFA *and the intron 1 polymorphism of *LTA*, in particular, have been associated with altered levels of circulating TNF-α [15,16]. A few of these polymorphisms have been also studied for determining susceptibility or resistance towards tuberculosis in several ethnic groups, the results of which have been inconclusive [17-26].

The aim of this study was to determine associations, if any, of potentially functional *TNFA *and *LTA *polymorphism(s), both individually and at the haplotype level, with tuberculosis in patients from North-India. In addition, we have attempted to explore whether any of these polymorphisms may be related to the severity and the associated features of the disease. We also attempt to correlate these polymorphisms with sTNF-α levels in the patients and the controls.

## Methods

### Study Subjects

185 unrelated tuberculosis patients (mean age 32.16 ± 13.8 years; male:female 0.42:0.58), who presented to the Medical Outpatient Department or were admitted at the All India Institute of Medical Sciences hospital, New Delhi, between 1996 and 2006 were recruited. The study was approved by the ethical committee of the hospital and informed consent was obtained from all participants. Patients with chronic illness such as cirrhosis of liver, chronic hepatitis, acute viral hepatitis, and/or gastrointestinal, renal, or cardiac diseases and those who were tested positive for HIV using ELISA were excluded during the preliminary evaluations.

#### Pulmonary tuberculosis

There were 149 patients in this group (Table 1). The diagnosis of pulmonary TB was based on the presence of acid-fast bacilli on sputum smear or *M. tuberculosis *on sputum culture. In patients with smear-positive TB, culture was not done unless multidrug- resistant TB was strongly suspected. Sputum cultures were done in all patients with smear-negative pulmonary TB. In patients with negative smears and cultures (N = 17), the diagnosis of TB was based on symptoms, chest radiographic infiltrates in the upper lobes, and clinical and radiographic response to antituberculosis drugs. In addition, high resolution computed tomography (HRCT) for the patients showed tree-in-bud appearance compatible with the clinical diagnosis of pulmonary TB. Also, all 149 pulmonary TB patients showed a good clinical and radiographic response to anti-TB treatment. The severity of the pulmonary lesions was graded by studying the chest radiographs following the guidelines of the American Thoracic Society [27].

**Table 1:** Clinical characteristics of the tuberculosis patients used for the study

	PTB	DTB	LNTB
**No. of patients**	149	11	25
**Age (years; ± SD)**	32.50 ± 13.3	34.36 ± 12.9	29.12 ± 11.6
**Sex (M:F)**	0.37:0.63	0.27:0.73	0.76: 0.24
**Sputum smear**	129	0	0
**Sputum culture**	47	0	0
**Lymph node FNAC**	ND	2	7
**Lymph node biopsy**	ND	3	20
**Pus smear**	ND	4	0
**Pus culture**	ND	6	0
**Positive TST response**	79	8	17
**Response to treatment**	149	11	25

Some patients in the PTB group had both smears and cultures positive while in the LNTB group, two patients had positive FNAC as well as biopsy positive. Similarly, in the DTB group 4 patients had both smear and cultures positive from the cold abscesses.

DTB: disseminated tuberculosis; PTB: pulmonary tuberculosis; LNTB = lymph node tuberculosis; FNAC= fine needle aspiration cytology

#### Disseminated tuberculosis

Focal disseminated tuberculosis (DTB) was diagnosed based on the criteria including i) clinical features suggestive of tuberculosis; ii) concurrent involvement of at least two non-contiguous organ sites of the body or involvement of the bone marrow; iii) microbiological and/or histopathological evidence of tuberculosis; (iv) evidence of marked improvement on antituberculosis treatment. The presence of all criteria was required for the diagnosis of disseminated tuberculosis and there were 11 patients in this group (Table 1). Of these, 2 patients had positive Ziehl-Neelsen (ZN) staining on fine needle aspiration cytology (FNAC) of the lymph nodes; 3 patients had histopathology of lymph nodes compatible with the diagnosis of TB; 4 patients had both positive ZN staining and *Mycobacterium tuberculosis *(*Mtb*) culture positivity in the pus from cold abscess and additional 2 patients had only *Mtb *culture positivity. All patients showed a good clinical response to anti-TB treatment.

#### Lymph node tuberculosis

Lymph node tuberculosis (LNTB) was diagnosed based on the following criteria i) clinical presentation compatible with tuberculosis; ii) fine needle aspiration cytology presenting evidence of tuberculosis with positive staining for acid-fast bacilli; iii) histopathological evidence of caseating granulomas on lymph node biopsy specimens; iv) evidence of marked improvement on antituberculosis treatment. The presence of i), and either ii) or iii) and iv) was required for the diagnosis of lymph node tuberculosis and there were 25 patients in this group (Table 1). Of these, 7 patients had positive ZN staining on FNAC and 20 had histopathology of the lymph nodes compatible with the diagnosis of tuberculosis of the lymph nodes (2 of these also had positive FNAC). All these patients showed a good clinical response to anti-TB treatment.

#### Normal Controls

Healthy Volunteers (referred to as normal controls, N = 155; mean age 28.16 ± 13.6 years; male:female 0.46:0.54) were randomly recruited from the general population with the same socio-economic status and ethnic background as that of the patients. All individuals gave informed consent and were screened negative for HIV infection and a family history of tuberculosis or any other related disease. In addition, these individuals had a negative tuberculin test (to exclude a possibility of a latent tuberculosis infection) and their chest radiographs as well as peripheral blood counts and blood chemistries were normal.

All patients with TB and normal controls recruited for the study represented a fairly homogeneous ethnic group of north Indian Hindu population from the states of Punjab, Haryana, Uttar Pradesh and Delhi. All these individuals come under the Indo-European linguistic group and belong to the same ethnic group (group 1) [28,29].

### Tuberculin Skin Test (TST)

The TST was done using 5 tuberculin purified protein derivative (PPD) units (5 TU Tuberculin PPD/0.1 ml; Span Diagnostics Ltd., India); and the indurations were measured after 48 hours. A cut-off of 10 mm was used for a positive TST.

### Serum TNF-α measurement

Serum TNF-α levels were determined for 107 tuberculosis patients and 112 normal controls using the OPTEIA™ ELISA kit (BD Biosciences), as per the manufacturer's instruction.

### Genotyping

Isolation of the genomic DNA from the peripheral blood leukocytes was carried out using the modified salting out procedure as described elsewhere [16]. Polymorphisms in the *TNF *genes, namely *TNFA-1031T > C*, *TNFA-863C > A*, *TNFA-857T > C*, *TNFA-308G > A*, *TNFA-238G > A *and the *LTA*_*Nco*I polymorphism were genotyped using primer pairs as described elsewhere [16]. Briefly, the *TNFA-1031T > C*, *TNFA-863C > A*, *TNFA-857T > C*, *TNFA-308G > A *and the *TNFA-238G > A *were studied using SNaPshot ddNTP Primer Extension Kit (Applied Biosystems, Foster City, USA) as per the manufacturer's instructions. To clean up the primer extension reaction, 1U of calf intestinal phosphatase (CIP) (New England Biolabs) was added to the reaction mixture and the mixture was incubated at 37°C for 1 hour, followed by 15 minutes at 72°C for enzyme inactivation. These samples were subsequently electrophoresed using the ABI Prism 3100 Genetic Analyzer as per the manufacturer's instructions. The results were analyzed using the GeneMapper software (Applied Biosystems, Foster City, USA). The *LTA_NcoI *polymorphism was assessed using NcoI restriction endonuclease digestion. The accuracy of genotyping was confirmed by direct sequencing of the DNA samples (N = 5) for all three respective genotypes for all the loci investigated.

### Statistical Analysis

The allele frequencies were calculated and agreement with Hardy-Weinberg equilibrium was tested using a χ^2 ^goodness-of-fit test for each locus. The association between two categorical variables was evaluated by likelihood ratio (LR) χ^2 ^and Fishers exact tests. All statistical tests performed were two-tailed. ANOVA was performed to test the effect of the polymorphisms and haplotypes on sTNF-α levels and F-ratio statistics were calculated to study the variation in sTNF-α levels in different groups. PHASE was used to infer haplotypes for each individual [30]. Clump22 software was used with 1,000,000 Monte-Carlo simulations to determine the primary difference in the haplotype frequencies [31]. Normal (T1) χ^2 ^values, calculated from the 2 × N raw data, and maximum (T4) χ^2 ^values, calculated by simulating 2 × N data followed by clumping into 2-by-2 tables to produce maximal chi-squared values, have been reported.

## Results

### Association of *TNF *polymorphisms with tuberculosis

The genotype frequencies for all the polymorphisms investigated did not deviate significantly from Hardy-Weinberg expectations in both the patient and the control populations (p > 0.05). Table 2 and Table 3 enlist the allele and the genotype frequencies for all the polymorphisms genotyped in the *TNF *genes, respectively. The comparison of the allele and genotype frequencies for all the loci investigated revealed no significant differences between the tuberculosis patients and controls (p > 0.05; Table 2 and Table 3, respectively).

**Table 2:** Allele frequencies of the polymorphisms investigated in the *TNFA *and *LTA *genes.

Polymorphism	Allele	Tuberculosis Patients (2N = 370)	Normal Controls (2N = 310)	FET p-value
***Nco*I**	**A**	270 (72.97)	244 (78.71)	0.09
	**G**	100 (27.03)	66 (21.29)	

***-1031***	**C**	136 (36.76)	110 (35.48)	0.79
	**T**	234 (63.24)	200 (64.52)	

***-863***	**A**	119 (32.16)	100 (32.26)	>0.99
	**C**	251 (67.84)	210 (67.74)	

***-857***	**C**	339 (91.62)	276 (89.03)	0.29
	**T**	31 (8.38)	34 (10.97)	

***-308***	**A**	36 (9.73)	27 (8.71)	0.69
	**G**	334 (90.27)	283 (91.29)	

***-238***	**A**	23 (6.22)	14 (4.52)	0.40
	**G**	347 (93.78)	296 (95.48)	

Numbers in parentheses indicate the frequency (%).

**Table 3:** Genotype frequencies of the polymorphisms investigated in the *TNFA *and *LTA *genes.

Polymorphism	Allele	Tuberculosis Patients (2N = 185)	Normal Controls (2N = 155)	**LR χ**^**2**^	p-value (df = 2)
***Nco*I**	**AA**	94 (50.81)	97 (62.58)	3.43	
	**AG**	82 (44.32)	50 (32.26)		0.18
	**GG**	9 (4.86)	8 (5.16)		

***-1031***	**CC**	25 (13.51)	20 (12.90)	0.13	
	**CT**	86 (46.49)	70 (45.16)		0.94
	**TT**	74 (40.0)	65 (41.94)		

***-863***	**AA**	17 (9.19)	17 (10.97)	0.53	
	**AC**	85 (45.95)	66 (42.58)		0.77
	**CC**	83 (44.86)	72 (46.45)		

***-857***	**CC**	154 (83.24)	123 (79.35)	NC	
	**CT**	31 (16.76)	30 (19.35)		NS
	**TT**	0 (0)	2 (1.29)		

***-308***	**AA**	3 (1.62)	2 (1.29)	NC	
	**AG**	30 (16.22)	23 (14.84)		NS
	**GG**	152 (82.16)	130 (83.87)		

***-238***	**AA**	2 (1.08)	0 (0)	NC	
	**AG**	19 (10.27)	14 (9.03)		NS
	**GG**	164 (88.65)	141 (90.97)		

Numbers in parentheses indicate the frequency (%).

df: Degrees of freedom; NC - Test not conducted as counts are less than 5 for a few categories; NS - Non-significant.

### Association of *TNF *polymorphisms in tuberculosis sub-groups

The severity of the pulmonary lesions was graded by studying the chest radiographs following the guidelines of the American Thoracic Society [27]; the patients were sub-grouped into minimal, moderately advanced and far advanced. Further, the patients were also sub-grouped on the basis of response to TST (negative/positive) and the presence/absence of cavitary lesions. However, none of the studied polymorphisms showed a significant association with any of the patient sub-groups (p > 0.05; data not shown).

### Association of *TNF *haplotypes with tuberculosis

**Figure 1:**
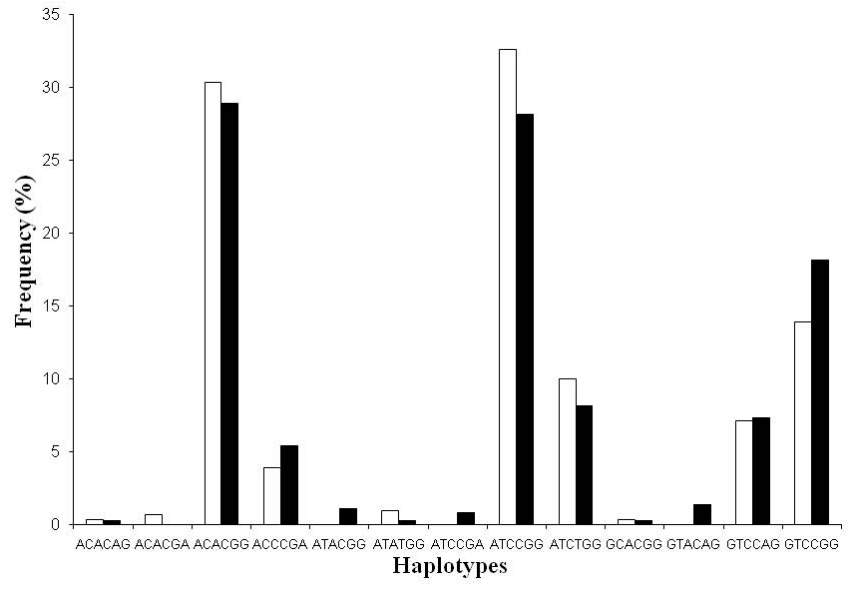
**The frequency distribution of the haplotypes of the *TNFA *and *LTA *genes**. The frequency distribution of the haplotypes of the *TNFA *and *LTA *genes in tuberculosis patients (dark bars) and unrelated controls (light bars). The haplotypes have been plotted on the *x*-axis and their respective relative frequencies (%) on the *y*-axis.

To study the combined effects of the six SNPs in the *TNF *genes, haplotypes were estimated by the statistical software PHASE. Of the 13 haplotypes observed in the 185 TB patients and 155 controls recruited for the study, only five haplotypes were represented at frequencies >5% (Figure 1). These accounted for >87% of the total haplotypes. As the count per haplotype was very low, Clump22 with 1,000,000 Monte-Carlo simulations was used to estimate differences in the haplotype frequencies in the patients and controls. However, no significant differences were observed in the haplotype counts in the two groups [normal (T1) χ^2 ^= 18.498, df = 12, P = 0.78; and maximum (T4) χ^2 ^= 7.825, P = 0.214]. Therefore, it may be concluded that haplotypes in the *TNF *genes were not associated with tuberculosis in the studied population.

### Association analyses of *TNF *polymorphisms with its serum levels

TNF-α levels were measured in the serum samples of 107 tuberculosis patients and 112 unrelated controls. A difference with borderline significance was observed in the serum TNF-α levels in the patients and the controls (F-ratio = 3.84, df = 1, p = 0.051); a higher levels of the cytokine was observed in the patients sera [log sTNF-α ± standard deviation (s.d.) = 1.89 ± 0.87] when compared with the control population [log sTNF-α ± s.d. = 1.65 ± 0.89].

Although, none of the investigated polymorphisms were found to affect the sTNF-α levels in both the patient and the control populations and in the patient sub-groups (p > 0.05), a few interesting observations were made. Of the 107 patients, 39 patients with positive TST showed higher log sTNF-α levels (2.10 ± 0.91) while 68 patients with negative TST showed lower log sTNF-α levels (1.75 ± 0.84) [F-ratio = 3.92, df = 1, p = 0.05]. Furthermore, when the patients were graded on the basis of severity into minimal severity, moderately advanced and far advanced sub-groups, it was observed that patients with minimal severity had lower log sTNF-α levels (1.54 ± 0.73) when compared to the patients with moderately advanced (1.86 ± 0.98) and far advanced severity (1.96 ± 0.71). However, none of these differences were statistically significant (p > 0.05). Additionally, no difference was observed in the log sTNF-α levels when patients were sub-grouped on the basis of presence/absence of cavitary lesions in the X-rays (p > 0.05).

Furthermore, when haplotypes (counts ≥ 2) were analyzed with respect to sTNF-α levels in the patient samples (case-only analysis), no significant difference were observed (p-value > 0.05), thereby negating the influence of any of the studied polymorphisms on sTNF-α levels in the study population.

## Discussion

TNF-α plays an important role in granuloma formation in tuberculosis. Previous studies have shown that mice deficient in TNF-α exhibit poorly formed granulomas with areas of extensive necrosis, resulting in widespread dissemination of *M. tuberculosis *and rapid death of animals [32]. Earlier data obtained from the rheumatoid arthritis patients treated with TNF-α antagonists showed that blocking TNF-α could lead to reactivation of tuberculosis [33]. All these points hint towards *TNFA *as an important candidate gene for tuberculosis.

The promoter region of the *TNFA *gene is highly polymorphic. In this case-control study, conducted in tuberculosis patients and normal controls recruited from Northern India, we focused on potential functional polymorphisms in the transcriptional region of *TNFA *and a *LTA_NcoI *(+252) polymorphism in the first intron of *LTA *gene a few of which have earlier been associated with TNF-α levels [15,16]. Individually, none of the polymorphisms showed any significant association with tuberculosis or sTNF-α levels. An earlier study carried out in *HLA-A*, *B *and *DR *typed pulmonary tuberculosis patients (N = 210) and healthy control subjects (N = 120) from South India also failed to detect any significant differences between the *LTA_NcoI *and *TNFA_-308 *and *TNFA_-238 *polymorphisms and tuberculosis [20]. These included active and cured tuberculosis patients as well as controls of Indo-dravidian origin, an ethnicity different from ours, recruited from North-India and belonging to the Indo-European linguistic group. Additionally, another recent study carried out in the north-Indian populations also failed to find any association of *TNFA *-308 promoter polymorphism with tuberculosis [34]. This study was conducted in ethnically matched 145 physician diagnosed tuberculosis patients and 211 normal healthy controls recruited from the state of Punjab from North India. However, in both these studies no attempts were made to correlate these and the other promoter polymorphisms individually and at the level of haplotype with sTNF-α levels. Also, the normal controls recruited for our study were tested negative by the TST test and had normal blood chemistries, peripheral blood counts and normal chest radiographs. Additionally, all patients and controls recruited for our study were HIV-negative. Our study also gains support from others which report a lack of association between *TNF *gene polymorphisms and tuberculosis in different populations (Table 4). On the other hand, a few studies reported association between the *TNFA_-308 *polymorphism and tuberculosis [22,35-37]. However, these differences in results could be attributed to ethnic variations that persist in different populations, study design and patient selection criteria. Additionally, the association observed in the later studies may be explained on the basis of linkage disequilibrium that exists between the *TNF *genes and *HLA *[38].

**Table 4:** Association studies of the *TNF *genes with tuberculosis.

S. No	Reference	Polymorphisms Investigated	Location	Population	Sample size	Results
1.	Selvaraj et al. [20]	*TNFA *-238, -308 and *LTA *(Nco I)	Promoter; Intron 1	South Indian	210 PTB patients and 120 normal controls (NC)	No association with pulmonary TB (PTB).

2.	Ates et al. [17]	*TNFA *-308 G/A, -238 G/A, -376 G/A	Promoter	Turkish	128 TB patients and 80 NC	No association with TB.

3.	Vejbaesya et al. [21]	*TNFA *+488, -238, and -308	Promoter	Thai	149 TB patients and 147 NC	No association with TB.

4.	Amirzargar et al. [37]	*TNFA *-308, G/A -238	Promoter	Iranian	41 PTB and 123 NC	-238 polymorphism was associated with pulmonary TB (p = 0.0009).

5.	Shaw et al. [42]	Linkage study	Markers across the genome	Northern Brazilian	98 pedigrees, with 704 individuals	Minor role of *TNFA *in control of TB.

6.	Correa et al. [35,36]	*TNFA *-308, G/A -238	Promoter	Columbia	138 TB and 419 NC	Association of *TNF1 *with TB (OR: 1.9, 95%C.I. 1.2-3.1, p = 0.02)

7.	Wu et al. [24]	*TNFA *-308	Promoter	Chinese	61 PTB patients and 122 PTB-free miners.	Lack of association with TB.

8.	Larcombe et al. [26]	*TNFA *-308	Promoter	Canadian Aboriginal	Three cohorts n = 61, 42 and 91 for Dené, Cree and healthy Caucasians, respectively	Lack of association with TB.

9.	Henao et al. [25]	*TNFA *-308	Promoter	Colombian	54 tuberculin-negative NC, 81 tuberculin-positive NC, 140 PTB patients, 30 with pleural TB and 20 with miliary TB	Lack of association with TB.

10.	Kumar et al. [34]	*TNFA *-308	Promoter	Indian	145 TB and 211 NC	Lack of association with TB.

11.	Oh et al. [18]	*TNFA *-308	Promoter	Korean	117 NC, 80 newly diagnosed TB patients and 65 patients with recurrent TB	*TNFA *gene does not affect differential TB susceptibility.

12.	Sharma et al. [Current]	*LTA_Nco*I; *TNFA *-238, -308, -857, -863, -1031	Intron 1; Promoter	North Indian	185 TB patients and 155 NC	Lack of association with TB.

PTB = pulmonary tuberculosis; NC = normal controls

Our study also failed to detect any effect of the *TNF *polymorphisms on sTNF-α levels in the tuberculosis patients. The results from previous *in vivo *and *in vitro *studies conducted for the *TNFA-308G > A *polymorphism have rather been conflicting. Whereas there are studies which report the minor *TNFA-308A *allele (allele 2) to be associated with higher inducible levels of gene transcription and TNF-α production [39,40], our earlier study in sarcoidosis patients found the G allele for this polymorphism to be associated with high sTNF-α levels [16]. It is also evident from the study by Knight et al that the *TNFA*-308 SNP does not regulate TNF-α levels and is not likely to be the functionally important SNP as previously hypothesized [41]. Thus, it may be possible that another SNP in LD is involved in this phenomenon. Further high resolution *HLA *typing data in addition to fine mapping of the *TNF *genes would be of help in resolving these problems.

To enhance our understanding about the contribution of these genetic variants further to tuberculosis, six-locus haplotypes were constructed and their distribution was compared in the patients and the control population. None of the haplotype showed significant association with tuberculosis in the study population.

## Conclusions

In summary, our findings suggest that the polymorphisms present in and around the *TNF *genes are unlikely to be the major risk factors for tuberculosis in Asian Indians. However, as the present study was performed with relatively small sample size, further studies with larger sample sizes would be necessary to elucidate the role of *TNF *polymorphisms in tuberculosis.

## Abbreviations

HLA: Human leukocyte antigen; LR: Likelihood ratio; LTA: Lymphotoxin α; PPD: Purified protein derivative; TB: Tuberculosis; TNF: Tumor necrosis factor; TST: Tuberculin skin test

## Competing interests

The authors declare that they have no competing interests.

## Authors' contributions

SS, BG and SKS conceived and designed the study. SS and JR were involved in collection of the data. SS, JR, BG and SKS were involved in the analysis and interpretation of the data. SS, BG and SKS were involved in preparation and critical revision of the manuscript. All authors have approved the final version of the manuscript.

## Pre-publication history

The pre-publication history for this paper can be accessed here:

http://www.biomedcentral.com/1471-2334/10/165/prepub
